# High-resolution estimates of the US population in fluvial or coastal flood hazard areas

**DOI:** 10.1038/s41597-025-05717-y

**Published:** 2025-08-07

**Authors:** Adam C. Gold, Ivy Steinberg-McElroy

**Affiliations:** 1 Environmental Defense Fund, Raleigh, NC USA; 2 Environmental Defense Fund, New York, NY USA

**Keywords:** Natural hazards, Hydrology

## Abstract

Flooding is the most common and damaging natural disaster in the United States (US), and understanding the number of people at risk of flooding is critical information for planning. The dataset presented here uses publicly available census and building footprint data to improve upon previous estimates of the number of people and housing units in fluvial or coastal flood hazard areas in the contiguous US. To calculate the population and housing unit estimates, the ratio of total residential building footprint area that intersects high flood hazard areas is multiplied by 2020 Decennial Census block counts. This flexible framework will allow the dataset to be updated over time and with additional flood risk datasets such as those that incorporate pluvial flooding. These high-resolution estimates of people and housing in fluvial or coastal flood hazard areas will provide valuable information to flood resilience planning efforts.

## Background & Summary

Flooding is the most costly and widespread natural disaster in the US, causing the 2023 equivalent of between $179.8 and $496.0 billion US dollars to homes and infrastructure each year^1^. The 2024 hurricane season saw disasters such as hurricanes Helene, Milton, and Beryl, which resulted $79.6 billion USD, $34.3 billion USD, and $7.2 billion USD in damages, respectively, and a combined 297 casualties^2^. There are ways to increase resilience and decrease the impacts of flooding - communities can proactively plan by understanding their flood risk, which is a combination of flood hazard and the vulnerability of people and property to that hazard^3,4^. Flood hazard is generally informed by hydrologic and hydraulic modeling, while vulnerability relies on an understanding of where people and property are located and how they would be impacted by and respond to flooding^5^.

Previous studies have estimated the number of people in the US that live in the floodplain^6–10^, but the precision of these estimates can be improved. A common methodology for estimates of the number of people at high risk of flooding in the US uses the Federal Emergency Management Agency (FEMA) special flood hazard area (SFHA), sometimes supplemented with an estimated SFHA^11^, as the combined measure of fluvial and coastal flood hazard. To estimate the population vulnerable to that flood hazard, studies have counted people as impacted based on a range of approaches that include intersecting a hazard layer with US Census boundaries or census-derived data. Studies have intersected flood hazard layers with census block centroids^12^ or scaled population by the proportion of census block area intersecting a flood hazard layer^6^, but these approaches have a spatial mismatch between buildings and flood hazard by not explicitly accounting for the location of buildings. Some studies have intersected flood hazard with medium-resolution gridded dasymetric population products^7,8,10,13^, but these gridded population products often rely on coarse land cover datasets that can lead to classification errors and resolution mismatches with flood hazard data. More recent studies have used the proportion of building footprints intersecting the flood hazard layer for each census block or block group^9,14^, but these methods either do not isolate residential buildings or rely on coarse-resolution input data that limits the precision of results. Most previous studies also do not account for uncertainty in census counts, though there may be significant error or privacy noise at smaller spatial scales^15^. The methods for estimating how many people live in high fluvial and coastal flood hazard areas have become more precise over time, but improvements are still needed to: (**1**) better isolate impacts to residential buildings, (**2**) estimate uncertainty using census data, and (**3**) use only publicly available datasets. It is important to address these gaps to provide accurate representations of fluvial and coastal flood risk that can inform policies that further decrease flood risk to people and property.

**Fig. 1:**
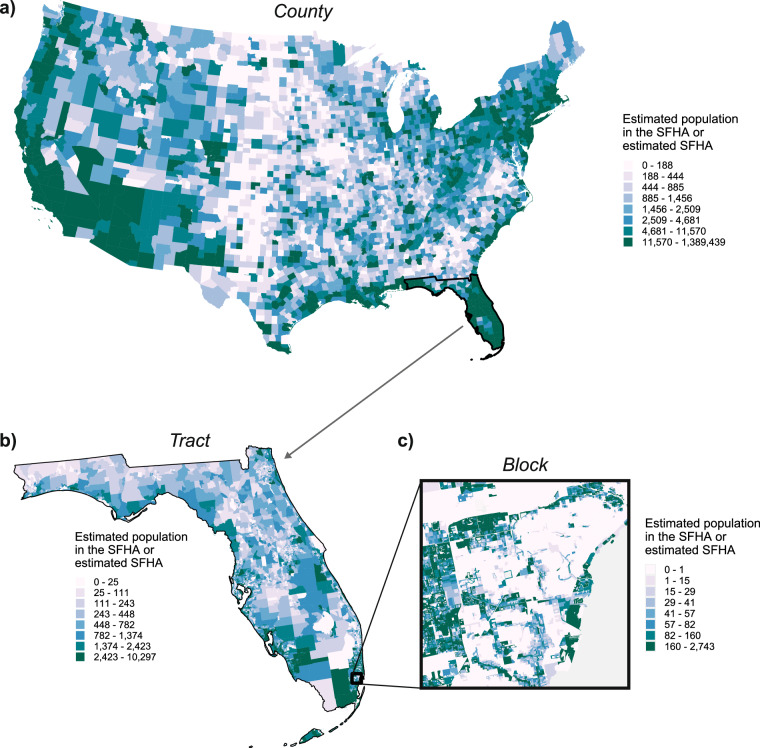
Estimated population living in the SFHA or estimated SFHA by (**a**) county for the contiguous US, (**b**) by census tract for the state of Florida, and (**c**) by census block for a subset of Miami, Florida.

This article introduces FloodPop (Fig. 1), an open-access dataset representing high-resolution estimates of the US population and housing units in areas subject to fluvial or coastal flooding down to the census block level in the contiguous US. FloodPop improves the precision of estimates of people and housing in high flood risk areas by more accurately identifying residential buildings, estimating confidence intervals using census data, and using only publicly-available datasets. FloodPop was created by scaling 2020 Census block populations, total housing units, and occupied housing units by the percent of residential building footprint area intersecting the FEMA SFHA or EPA estimated SFHA in each census block. Building footprint areas are calculated based on a novel “residential-or-not” classified building footprint dataset created by combing multiple publicly available building footprint datasets. The FloodPop dataset is provided in both a tabular and geospatial format at the block, tract, county, and state level, allowing it to be combined with other census data and indices (Fig. 1).

Based on this new dataset, 24.4 million people (90% CI: 19.2–30.7 million), or 7.4% (5.8–9.3%) of the population in the contiguous US, are estimated to live in either the FEMA SFHA or estimated SFHA (Table 1, Fig. 1). For housing, 11.8 million housing units in the contiguous US are estimated to be in fluvial or coastal flood hazard areas, and 9.8 million housing units are occupied (90% CI: 7.7–10.9 million). The estimate of the population at risk of fluvial or coastal flooding based on just the SFHA (13.7 million people, Table 1) or best-available SFHA (14.8 million people, Table 1) is comparable to estimates from previous studies for the contiguous US (11.12–15 million people^10,14,16^). The estimate of the population at risk of flooding based on either SFHA, 24.3 million people (Table 1), is notably higher than those previous studies that rely solely on the mapped SFHA, but much lower than the 40.8 million people estimated to be at risk due to fluvial, coastal, or pluvial flooding and a medium-scale dasymetric population product^10^.

**Table 1:** Total counts of population and housing units in the contiguous US, and estimates of population and housing units in the SFHA, best-available SFHA (FEMA or estimated SFHA), or either SFHA for the contiguous US.

Category	Population	Total Housing Units	Occupied Housing Units
Total	329,260,619	139,611,470	126,058,165
SFHA	13,695,823 (10,896,742–17,077,238)	6,800,897	5,543,505 (4,378,576–6,176,456)
Best-available SFHA	14,802,156 (11,644,010–18,658,967)	7,343,881	5,975,735 (4,669,884–6,685,790)
Either SFHA	24,377,689 (19,207,613–30,676,565)	11,835,254	9,837,903 (7,724,548–10,928,140)

Upper and lower bounds represent 90% confidence intervals (CI).

FloodPop provides critical open-access information for flood resilience efforts, and the framework and input data can be adapted for higher resolution flooding data, additional climate-related hazards, and non-census boundaries. This dataset could be utilized and adapted by multiple end users, for example by local municipalities, to identify a baseline level of flood risk and use it to inform river basin studies, hazard mitigation plans, and floodplain ordinances. “Residential-or-not” building footprints could also be used to inform zoning policies, compare risk to residential vs. commercial/industrial buildings, and identify hotspots of risk for other climate-related hazards. FloodPop uses the best-available national data on fluvial and coastal flood hazard, but the FEMA SFHA does not capture all potential flood risk^9,10,17^. As 2-dimensional flood modeling, which captures fluvial (riverine), coastal, and pluvial (rainfall-driven) flooding, becomes more common, the FloodPop framework and novel classified building footprint dataset can be used to generate estimates of the population and housing units impacted based on 2-D model outputs. Also, the framework and code used to create this dataset can be used with other higher-resolution local input data of hazards and can be adapted to additional non-census boundaries such as watershed boundaries. This dataset and methods framework can improve flood resilience efforts at multiple scales by helping identify hotspots of flood risk with greater spatial precision, error estimation, and adaptability to future flood hazard data.

## Methods

### Overview

**Fig. 2:**
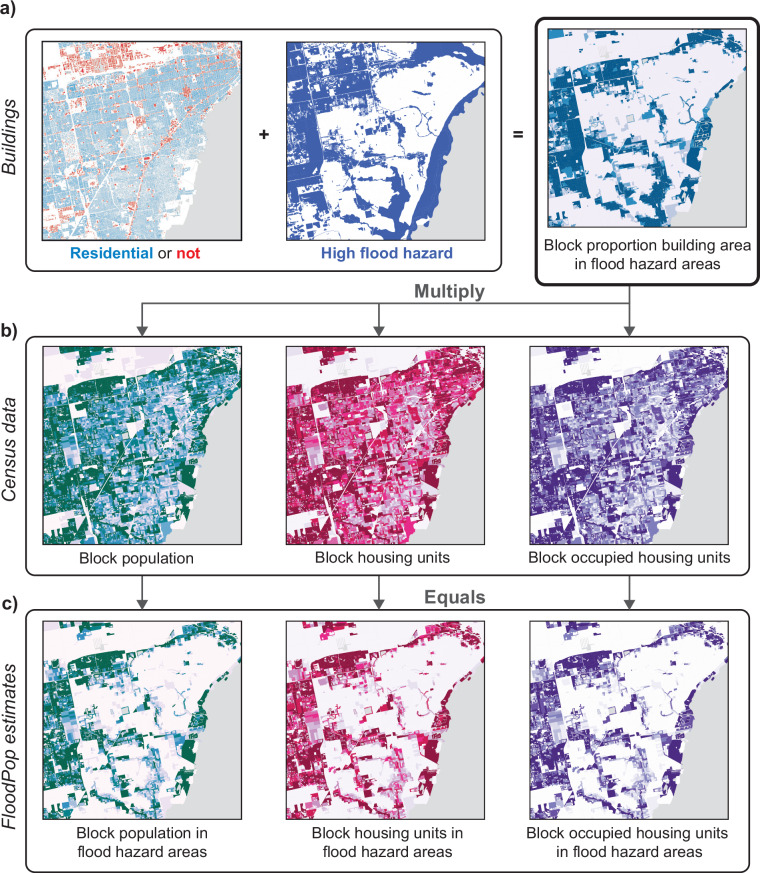
Schematic of the workflow to create FloodPop and related datasets.

The FloodPop dataset was created by scaling 2020 Census block population and housing unit values by the proportion of census block residential building area intersecting fluvial and coastal flood hazard locations (Fig. 2). The estimates provided here include confidence intervals based on calculated confidence intervals of the underlying census block counts. The novel building footprint dataset presented here and used to scale census data combines the best-available open-source building footprints that lack a standard building type classification with the best-available open-source building type classifications that can lack in spatial precision. While this article presents the FloodPop dataset, the underlying building footprint dataset and methodological framework could be used to estimate the number of people and housing units affected by other climate-related hazards where the footprint of impact is available and requires high spatial precision to estimate impacts to residential structures.

### Building footprints

Buildings footprints from the 2024-11-13.0 release of Overture data^18^ were downloaded for the contiguous US by state. This dataset combines building footprints from multiple open sources, in order the following order: OpenStreetMap (OSM)^19^, Esri Community Maps^20^, Google Open Buildings (>90% precision)^21^, Microsoft^22^, and Google Open Buildings (<90% precision). Buildings for each state were projected and saved to a feature class. Most Overture building footprints for the Contiguous US come from either OSM or Microsoft, and in some cases, especially rural areas, some building footprints might not be captured. To address this, we used the 2022 version of the National Structures Inventory (NSI)^23^ to find potential missed building footprints in the Overture dataset. The NSI is a point dataset, but most points lie at the centroid of a building footprint from either the US release of Microsoft building footprints^24^ or FEMA’s USA Structures^25,26^. To identify potential missed building footprints (false negatives), NSI points whose location was derived from a parcel or building footprint (Source! = “X”) and did not overlap an Overture building footprint were identified. If the NSI points were related to a Microsoft building footprint (i.e., US release), the Microsoft building footprint was checked for overlap with Overture building footprints, and if there was no overlap, the Microsoft footprint was added to the Overture dataset. The same process was repeated for NSI points that did not overlap an Overture building footprint but were related to USA structures footprints.

### Footprint classification

**Fig. 3:**
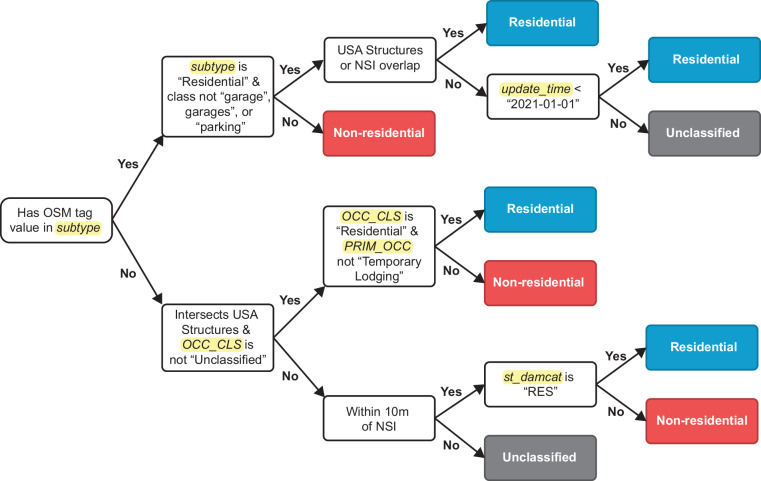
Flowchart for “residential” and “non-residential” classification of building footprints. Column names in the building footprint dataset are highlighted in yellow.

The amended Overture building footprint dataset was then classified as “residential”, “not residential”, or “unclassified” using, in order, OSM tags, USA Structures, and the 2022 NSI (Fig. 3). A “residential” classification is defined for this dataset as a residence where people live, so outbuildings, sheds, and garages on residential parcels are ideally classified as “non-residential” or “unclassified”, but sometimes spatial offset from USA structures would intersect outbuildings. Overture building footprints already contain columns representing OSM tags (i.e., subtype and class), and USA Structures and NSI points were spatially joined to the building footprint dataset. If a building contained OSM tag information, it was classified as “residential” if the “subtype” value was “residential” and was not a garage or parking; otherwise, it was classified as “non-residential” (Fig. 3). To improve the temporal match between the building footprints and 2020 Census data, “residential” buildings whose only classification data source was OSM and “update_time” was 2021 or later were labeled “unclassified”, as these buildings were likely built after 2020 and would not align with the 2020 Census data (Fig. 3). If a building did not contain OSM tag information but intersected a USA Structures polygon with a classification other than “unclassified”, the column “OCC_CLS” was used to determine the residential-or-not classification (Fig. 3). After that, any buildings without a classification were assigned a “residential” classification if they were within 10 m of an NSI point that was “residential” or “non-residential” if not (Fig. 3). Most building footprints were classified using USA Structures (77.65%), followed by OSM (14.73%), and the NSI (7.63%) (Supplementary Table 1).

To remove any potential building footprint overlaps and avoid double counting of building area, building footprints were “unioned” and overlaps were removed by finding identical geometries and deleting all but one copy. In removing overlaps, buildings that were classified as “residential” were prioritized, followed by prioritization by most recent “update_time”. Overlapping buildings were extremely uncommon but typically, if not always, due to multiple instances of the same building in the OSM-sourced building footprints. The “flattened” building footprint geometries were then validated using Open Geospatial Consortium standards.

### Identifying buildings in high flood hazard areas

The FEMA Special Flood Hazard Area (SFHA)^27^ and estimated SFHA^11^ were used to denote fluvial or coastal flood hazard areas. FEMA is the federal agency responsible for coordinating the federal response to disasters and the FEMA SFHA is a regulatory product and is created through 1-dimensional flood modeling that captures fluvial (i.e., riverine) flooding along the study reach and coastal flooding along shorelines. Approximately 40% of the Contiguous US does not have FEMA flood maps and therefore do not have a mapped SFHA^11^. The estimated SFHA uses coarser resolution input data (30 m resolution) than the FEMA SFHA, but it fills the data gap where the SFHA has not been mapped. The estimated SFHA can also capture fluvial flood risk in smaller tributaries higher in the watershed, complementing the mapped SFHA because FEMA studies typically do not include these headwater areas^11^. One limitation of the estimated SFHA to note is that it is based on physical attributes only and may identify areas as high flood risk when the true risk is lower due to interventions such as levees – so it may overestimate risk in these areas^11^. An additional limitation is that FEMA flood maps do not capture pluvial, or rainfall-induced, flood risk, which is becoming increasingly prevalent in urban areas due to increased rainfall intensity, increased impervious surfaces and infrastructure, and limitations of urban drainage systems. While this analysis does not capture pluvial flooding, limiting its applicability to capture the full depth of flood risk, future work can be done to add pluvial modeling in areas where those data exist. The estimated SFHA was used to supplement the FEMA-modeled SFHA, and three definitions of “high flood hazard” areas were used: 1) the FEMA-modeled SFHA, 2) the “best-available” SFHA, where the estimated SFHA is used outside the footprints of FEMA studies, and 3) either SFHA.

Buildings were overlayed with the SFHA and estimated SFHA, and a column for each SFHA layer was added to the building footprint dataset. If a building interested an SFHA layer, the corresponding column was populated with a value of “1”. Buildings were also overlayed with footprints of the FEMA studies so that the “best available” SFHA could be created by using the SFHA as the flood hazard layer where FEMA studies had occurred and the estimated SFHA where no FEMA studies had occurred. Preserving the intersection of each building with the SFHA, estimated SFHA, and flood study footprints allows for calculations of population and housing information in high flood hazard areas using either one of, both, or best-available flood hazard layers. Figures and values shown in this article use both flood hazard layers.

### Estimating population and housing units in fluvial or coastal flood hazard areas

To estimate the number of people and housing units in fluvial and coastal flood hazard areas, census block information from the 2020 Decennial Census was multiplied by the proportion of each block’s “residential” building area intersecting the FEMA or estimated SFHA. The 2020 Decennial Census was used because block-level information is the highest spatial resolution reported by the Census Bureau and is only collected during a decennial census. The 2020 Decennial Census has known coverage issues, like under-counting some race or ethnic groups (i.e., Black or African American and Hispanic) and overcounting others (i.e., Asian and Non-Hispanic White)^15^. The Census Bureau also utilized a new disclosure avoidance system (DAS) for the 2020 Census, and this DAS used a framework called “differential privacy” that adds noise so that information about individuals cannot be re-created^28^. Both coverage issues and the DAS add uncertainty to the estimates of the number of people and housing units in high flood hazard areas, but we assume that using census blocks, rather than larger block groups or tracts where there might be less relative noise or error, is a more appropriate spatial scale by which to apportion population and housing information based on building footprints.

We addressed uncertainty in census block counts due to the DAS by estimating confidence intervals for census block population and occupied housing unit counts. The Census Bureau published microdata files and resources that can be used to estimate confidence intervals for 2020 Census information using approximate Monte Carlo Simulation methods^29,30^. Following these methods, 90% confidence intervals for total population and occupied housing units at the census block level were estimated. Counts of total housing units by census block are not impacted by the DAS, so no confidence intervals were calculated for this variable. Same as the census block counts, the confidence intervals for the estimates of people and housing units in high flood hazard areas presented here are 90% confidence intervals.

## Data Records

The FloodPop dataset and underlying classified building footprints for the contiguous US are available on figshare^31^: 10.6084/m9.figshare.28681502. Summaries of FloodPop estimates by state are provided in Supplementary Tables 2–4. Results are provided in the following folder structure, with descriptions of the contents of each folder

**fp_summaries**: FloodPop results in tabular and geospatial formats.

- *blocks_by_state.gdb*: An Esri file geodatabase containing a feature class of FloodPop results at the census block level for each state. State abbreviations are at the beginning of the feature class name.
- *cartographic.gdb*: An Esri file geodatabase containing feature classes representing cartographic tract, county, and state level FloodPop estimates. The feature classes should be used for visualization but not used for analysis.
- *summaries.gdb*: An Esri file geodatabase containing feature classes representing tract, county, and state level FloodPop estimates. The feature classes can be used for analysis.
- *summary_csvs*: Comma Separated Value (CSV) files containing FloodPop estimates for census blocks, tracts, counties, and states. This folder also contains a CSV that summarizes the sources of building classifications by state.

**building_footprints.gdb**: An Esri file geodatabase containing classified building footprint feature classes for each state used to create FloodPop estimates. Each footprint contains information on building classification, presence within the SFHA, estimated SFHA, and FEMA study footprint, and census block. State abbreviations are at the beginning of the feature class name.

**building_footprint_dfs**: Folder containing a tabular version (Parquet format) of building footprints for each state. State abbreviations are at the beginning of the file name.

**validation:** Folder containing geodatabases used for the presented validation exercises.

## Technical Validation

### Building footprint classifications

The classification of high-quality Overture Maps building footprint data used a range of data sources, so these classifications were validated using three local parcel datasets – Mecklenburg County, NC^32^, Sacramento County, CA^33^, and Miami-Dade County, FL^34,35^. Parcels for each validation area were converted to a uniform classification of “residential”, “non-residential”, or “unclassified” (See Supplementary Materials). The “unclassified” parcels and parcels where the largest building was “unclassified” were not considered for this validation exercise. For all other parcels, the “residential” or “non-residential” classification of the largest building footprint on each parcel was compared to the parcel’s classification.

Parcels were used instead of building footprints due to the building classification’s workflow classifying most small outbuildings (e.g., sheds and garages) as “unclassified” or “non-residential”. Many local building footprint datasets have high-resolution detail and classify even the smallest buildings on residential properties as “residential”. While the use of local parcel data is effective for evaluating the broad classifications, a limitation is that on large parcels with many buildings, such as apartment complexes or shopping centers, only the largest building on the parcel is evaluated. Another limitation of the validation workflow is that only one of the parcel datasets explicitly represents the year 2020 (i.e., Mecklenburg County), and given that the building classification data roughly represents pre-2020 conditions, some misclassifications may occur given the mismatch in time between datasets. To exclude “unclassified” parcels and buildings from the validation workflow, any building that intersected a parcel with a classification of “None” was re-classified as “None” and vice-versa.

In total, the validation datasets comprise over 1.3 million parcels, which intersect with 1.5 million building footprints totaling over 435 square kilometers (Table 2). Most parcels (88.3–92.8%) and buildings (79.9–88.8%) were classified as residential (Table 2). The residential building footprint area ranged from 61.5% (Mecklenburg County) to 74.9% (Sacramento County) of all building footprint area (Table 2), illustrating the importance of differentiating between residential and non-residential buildings for scaling population information.

**Table 2:** Information about local parcel datasets and overlapping building footprints.

Location	Classification*	Parcels with a building	Buildings	Building area (km^2^)
Mecklenburg County, NC	Residential	290,750	321,100	64.5
	Non-residential	12,073	29,444	33.2
	None	26,636	51,358	7.2
Miami-Dade County, FL	Residential	481,880	491,774	126.2
	Non-residential	32,046	48,853	52.6
	None	19,025	54,859	8.8
Sacramento County, CA	Residential	406,763	470,899	106.8
	Non-residential	18,457	32,104	31.1
	None	13,232	27,155	4.7

*Classifications for parcels are derived from the local classifications, while classifications for the buildings are derived from the workflow presented in this article.

Similar to previous studies on building type classifications^36,37^, the metrics of precision, recall, and F1 scores were used to evaluate the quality of the residential-or-not classifications applied to building footprints^38^. Precision measures the proportion of true positive classifications compared to all positive classifications, so in this context, it would represent the proportion of parcels classified as “residential” that are truly “residential”. Recall measures the proportion of all positive classifications that were identified, so in this context, it would represent the proportion of all “residential” parcels that were classified as such. F1 scores are the harmonic mean of precision and recall.

The results of the validation with local parcel data show that the residential-or-not building classifications perform well (Table 3). This study has a greater focus on residential classifications, and this class performs better than the non-residential class (i.e., residential F1 score >0.99, non-residential F1 score >0.88). The agreement between the building footprint classifications and local parcel data shows that residential parcels are being correctly identified. Most of the underlying classification data for the three sample locations are from USA Structures, which incorporates a national dataset of parcel information.

**Table 3:** Precision, recall, F1 scores, and average F1 scores between classified building footprints and local parcel datasets.

Location	Classification	Precision	Recall	F1 score	Average F1 score
Mecklenburg County, NC	Residential	1.00	1.00	1.00	0.98
	Non-residential	0.94	0.98	0.96	
Miami-Dade County, FL	Residential	0.99	1.00	0.99	0.95
	Non-residential	0.93	0.87	0.90	
Sacramento County, CA	Residential	1.00	0.99	1.00	0.94
	Non-residential	0.88	0.89	0.88	

### Building footprints as a proxy for housing

**Fig. 4:**
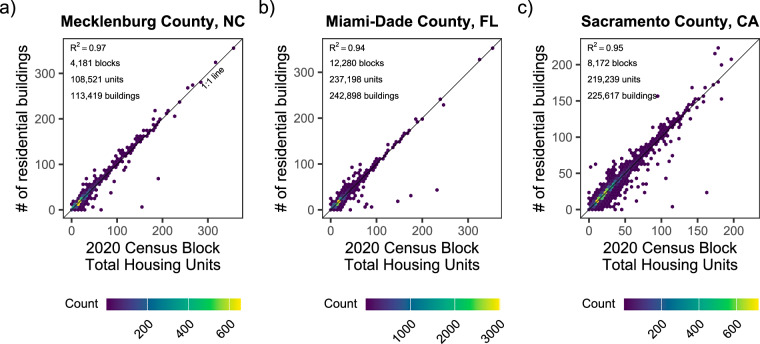
Comparisons of the estimated number of residential buildings from building footprints and total housing units for census blocks containing only “single-family” residential parcels.

To further validate the use of building footprints, we tested the assumption that building footprints can serve as a reasonable proxy by which to distribute population and housing unit information at the block level. To do this, we compared the number of “residential” classified buildings in census blocks to census measurements of total housing units. We focused only on census blocks where all the parcels in a census block were “single family” residential, allowing us to test if one housing unit equaled one “residential” building. These select census blocks represented approximately half of the census blocks with residential buildings in each location: Mecklenburg: 4,181/9,289 (45%), Miami-Dade County, FL: 12,280/24,746 (49.6%), Sacramento County, CA: 8,172/15,376 (53.1%) (Fig. 4).

In those select “single family” residential census blocks within the three validation locations, the relationships between the number of housing units and number of “residential” buildings in census blocks was strong (Fig. 4). All three validation locations exhibited a linear and positive 1:1 relationship between the number of “residential” buildings and total housing units (R^2^: 0.94–0.97, Fig. 4). Limited information was available for larger multi-family buildings, but census blocks consisting only of duplexes in Miami-Dade, FL and Sacramento County, CA yielded similarly strong and positive 1:2 relationships (i.e., 1 building for two housing units, Supplementary Figure 1). Overall, 24,633 census blocks were evaluated for this validation exercise, consisting of 564,958 housing units and 581,934 “residential” buildings. The results support the assumption that the “residential” building footprints can be reasonably used to spatially distribute census block population and housing information.

Importantly, the methodology to create FloodPop uses the ratio of building area intersecting fluvial or coastal flood hazard areas, rather than the ratio of building counts, to scale census block information. Comparing building area and population estimates across census blocks is complicated by inherent differences in house design and population density, which is why the validation exercise utilized only select census blocks to test the relationship between number of buildings and housing units, which we would expect to be a 1:1 relationship. To use building area intersecting fluvial or coastal flood hazard areas to scale census block information, we also assume that residential buildings within the same census block have the same population density (i.e., population per unit building footprint area). This assumption may not be accurate in census blocks where there are large differences in residential housing types and could bias results in those areas. To better understand potential impacts of this assumption, we used the three validation datasets to calculate the share of residential buildings that are classified as single family in each census block (Supplementary Figure 2). Our analysis of the three validation areas shows that most census blocks in these areas are either fully single-family homes or fully other types of denser housing (70–87%), so our assumption of constant population per unit building footprint area is reasonable given that most census blocks in our validation areas contain similar types of housing. To our knowledge, there is no publicly available information to ground-truth the scaling of census block data by building area, as this would require building-level population data. We suggest that the methodology used for FloodPop may improve accuracy compared to building counts by assuming that relatively larger footprint buildings in the same census block have more residents. In the future, incorporating building height and other building properties to move from footprint area-based to building volume-based population estimates would likely improve the accuracy of population estimates, especially in the share of census blocks with diverse housing types.

## Supplementary information

Supplementary Materials

## Data Availability

Code used to produce the FloodPop dataset is freely available on GitHub (https://github.com/acgold/floodpop) and is archived on Zenodo^39^.

## References

[1] *Flooding Costs the U.S. Between $179.8 and $496.0 Billion Each Year*. https://www.jec.senate.gov/public/_cache/files/51a2f463-5eab-4e8f-a5cf-202cc2214a0b/jec-report-on-economic-cost-of-flooding-update.pdf (2024).

[2] 2024: An active year of U.S. billion-dollar weather and climate disasters | NOAA Climate.gov. https://www.climate.gov/news-features/blogs/beyond-data/2024-active-year-us-billion-dollar-weather-and-climate-disasters (2025).

[3] Kron, W. Flood Risk = Hazard • Values • Vulnerability. *Water International* **30**, 58–68 (2009).

[4] Vojtek, M. & Vojteková, J. Flood hazard and flood risk assessment at the local spatial scale: a case study. *Geomatics, Natural Hazards and Risk* **7** (2016).

[5] Cutter, S. L., Boruff, B. J. & Shirley, L. Social Vulnerability to Environmental Hazards*. *Social Science Quarterly* **84** (2003).

[6] Barnard, P. L. *et al*. Projections of multiple climate-related coastal hazards for the US Southeast Atlantic. *Nat. Clim. Chang*. 1–9 10.1038/s41558-024-02180-2 (2024).

[7] Bousquin, J. & Hychka, K. A Geospatial Assessment of Flood Vulnerability Reduction by Freshwater Wetlands–A Benefit Indicators Approach. *Front. Environ. Sci*. **7** (2019). 10.3389/fenvs.2019.00054 PMC8312689 34316489

[8] Qiang, Y. Disparities of population exposed to flood hazards in the United States. *Journal of Environmental Management* **232**, 295–304 (2019). 30481643 10.1016/j.jenvman.2018.11.039

[9] Titus, J. G. Population in floodplains or close to sea level increased in US but declined in some counties—especially among Black residents. *Environ. Res. Lett.* **18**, 034001 (2023). 40092849 10.1088/1748-9326/acadf5 PMC11908447

[10] Wing, O. E. J. *et al*. Estimates of present and future flood risk in the conterminous United States. *Environ. Res. Lett.* **13**, 034023 (2018). 10.1088/1748-9326/aaac65 PMC11977400 40201223

[11] Woznicki, S. A., Baynes, J., Panlasigui, S., Mehaffey, M. & Neale, A. Development of a spatially complete floodplain map of the conterminous United States using random forest. *Science of The Total Environment* **647**, 942–953 (2019). 30180369 10.1016/j.scitotenv.2018.07.353 PMC8369336

[12] Logan, T. M., Anderson, M. J. & Reilly, A. C. Risk of isolation increases the expected burden from sea-level rise. *Nat. Clim. Chang.* **13**, 397–402 (2023).

[13] Swanwick, R. H. *et al*. Dasymetric population mapping based on US census data and 30-m gridded estimates of impervious surface. *Sci Data* **9**, 523 (2022). 36030258 10.1038/s41597-022-01603-z PMC9422266

[14] Huang, X. & Wang, C. Estimates of exposure to the 100-year floods in the conterminous United States using national building footprints. *International Journal of Disaster Risk Reduction* **50**, 101731 (2020).

[15] U. S. Government Accountability Office. 2020 Census: Coverage Errors and Challenges Inform 2030 Plans. https://www.gao.gov/products/gao-25-107160 (2024).

[16] NYU Furman Center. *Population in the U.S. Floodplains*. https://furmancenter.org/files/Floodplain_PopulationBrief_12DEC2017.pdf (2017).

[17] Sanchez, G. M. *et al*. The safe development paradox of the United States regulatory floodplain. *PloS One* **19** (2024). 10.1371/journal.pone.0311718 PMC11687735 39739663

[18] Overture Maps Foundation. Overture Maps Building Footprints. v2024-11-13.0 overturemaps.org (2024).

[19] OpenStreetMap Foundation. OpenStreetMap. https://www.openstreetmap.org/#map=5/38.01/-95.84 (2024).

[20] Esri. Esri Community Maps Program. https://communitymaps.arcgis.com/home/ (2024).

[21] Sirko, W. *et al*. Continental-Scale Building Detection from High Resolution Satellite Imagery. Preprint at 10.48550/arXiv.2107.12283 (2021).

[22] Microsoft. GlobalMLBuildingFootprints. https://github.com/microsoft/GlobalMLBuildingFootprints (2024).

[23] USACE. National Structure Inventory. 2022 https://www.hec.usace.army.mil/confluence/nsi/technicalreferences/2022 (2024).

[24] Microsoft. USBuildingFootprints. v2.0 https://github.com/microsoft/USBuildingFootprints (2018).

[25] FEMA. USA Structures Data. https://disasters.geoplatform.gov/USA_Structures/.

[26] Yang, H. L. *et al*. A baseline structure inventory with critical attribution for the US and its territories. *Sci Data* **11**, 502 (2024). 38755153 10.1038/s41597-024-03219-x PMC11099081

[27] National Flood Hazard Layer. Federal Emergency Management Agency https://www.fema.gov/flood-maps/national-flood-hazard-layer (2024).

[28] U.S. Census Bureau. Disclosure Avoidance for the 2020 Census: An Introduction. *Census.gov* https://www.census.gov/library/publications/2021/decennial/2020-census-disclosure-avoidance-handbook.html (2021).

[29] Ashmead, R., Hawes, M. B., Pritts, M., Zhuravlev, P. & Keller, S. A. An Approximate Monte Carlo Simulation Method for Estimating Uncertainty and Constructing Confidence Intervals for 2020 Census Statistics (2024).

[30] Estimating Confidence Intervals for 2020 Census Statistics Using Approximate Monte Carlo Simulation (2020 Census Production Run). https://registry.opendata.aws/census-2020-amc-mdf-replicates/?utm_campaign=20241024cnmps1&utm_medium=email&utm_source=govdelivery.

[31] Gold, A. C. & Steinberg-McElroy, I. High-resolution estimates of the US population in high fluvial flood hazard areas. *figshare*10.6084/m9.figshare.28681502. 10.1038/s41597-025-05717-y PMC12331959 40774974

[32] Mecklenburg County, NC. Mecklenburg County, NC parcel data. https://mecklenburgcounty.hosted-by-files.com/OpenMapping/Parcel%20Data%20Archive/Taxdata_2020.zip.

[33] Sacramento County GIS. Sacramento County, CA parcels. https://data-sacramentocounty.opendata.arcgis.com/datasets/sacramentocounty::parcels/about.

[34] Miami-Dade County, FL. Miami-Dade County, FL parcels. https://gis-mdc.opendata.arcgis.com/datasets/MDC::parcel/about.

[35] Miami-Dade County, FL. Miami-Dade County land use. https://gis-mdc.opendata.arcgis.com/datasets/MDC::land-use/about.

[36] Atwal, K. S., Anderson, T., Pfoser, D. & Züfle, A. Predicting building types using OpenStreetMap. *Sci Rep* **12**, 19976 (2022). 36404337 10.1038/s41598-022-24263-w PMC9676186

[37] F. de Arruda, H. *et al*. An OpenStreetMap derived building classification dataset for the United States. *Sci Data* **11**, 1210 (2024). 39521802 10.1038/s41597-024-04046-w PMC11550320

[38] Sokolova, M. & Lapalme, G. A systematic analysis of performance measures for classification tasks. *Information Processing & Management* **45**, 427–437 (2009).

[39] Gold, A. acgold/floodpop: v1.0. *Zenodo* v1.0 10.5281/ZENODO.15096915 (2025).

