# Supplementary material for: High-resolution estimates of the US population in fluvial or coastal flood hazard areas
Source: Sci Data. 2025 Aug 7;12:1377. doi: 10.1038/s41597-025-05717-y (PMC12331959; doi:10.1038/s41597-025-05717-y)
Supplement: Supplementary file 1 — Supplementary Materials [file 41597_2025_5717_MOESM1_ESM.pdf]

Supplementary Materials

High-resolution estimates of the US population in fluvial or coastal flood hazard areas

Adam C. Gold[1*] (ORCID: 0000-0002-0921-0384)
Ivy Steinberg-McElroy[2]

[1]Environmental Defense Fund, Raleigh, NC
[2]Environmental Defense Fund, New York, NY
*Corresponding email: agold@edf.org

Sources of classification data

Supplemental Table 1. Data sources of classifications by building classification and split by percentage of building counts and area. "USA" denotes the USA Structures dataset, "OSM" denotes the OpenStreetMap dataset, and "NSI" denote the National Structures Inventory.

	Source (% of total count)				Source (% of total area)			
**Classification**	**USA**	**OSM**	**NSI**	**Count**	**USA**	**OSM**	**NSI**	**Area (sq km)**
Residential	77.65	14.73	7.63	121,018,721	78.23	16.11	5.66	23,624.23
Non-residential	68.06	24.71	7.23	16,385,479	64.2	30.62	5.18	9,805.07
Unknown	-	-	-	20,282,137	-	-	-	2,649.42

State results

Supplementary Table 2. Total population in contiguous US states and estimates of population in the SFHA, estimated SFHA, or either SFHA in contiguous US states.

State	Total population	Population in SFHA	Population in best-available SFHA	Population in either SFHA
Florida	21,538,187	4,198,737 (3,563,231 - 4,922,432)	4,198,763 (3,563,250 - 4,922,465)	4,977,782 (4,227,433 - 5,839,762)
California	39,538,223	1,204,293 (981,221 - 1,460,634)	1,204,294 (981,221 - 1,460,637)	2,596,280 (2,133,248 - 3,127,822)

Texas	29,145,505	1,373,267 (1,092,441 - 1,710,567)	1,632,346 (1,288,043 - 2,048,143)	2,473,497 (1,987,394 - 3,058,322)
Louisiana	4,657,757	910,929 (728,968 - 1,123,036)	976,780 (779,417 - 1,208,260)	1,225,962 (984,038 - 1,509,968)
New York	20,201,249	639,069 (552,149 - 737,786)	810,054 (671,460 - 972,729)	1,160,219 (956,664 - 1,398,553)
Pennsylvania	13,002,700	292,073 (196,756 - 410,515)	292,073 (196,756 - 410,515)	679,706 (468,399 - 937,978)
New Jersey	9,288,994	495,437 (398,853 - 607,738)	508,079 (409,375 - 622,936)	668,458 (537,948 - 820,471)
Michigan	10,077,331	178,237 (138,733 - 224,737)	191,055 (147,272 - 243,470)	547,028 (430,208 - 685,394)
Ohio	11,799,448	205,430 (149,334 - 273,960)	211,845 (153,236 - 283,546)	543,007 (402,949 - 712,415)
Arizona	7,151,502	146,698 (110,492 - 191,302)	146,698 (110,492 - 191,302)	496,845 (389,912 - 623,965)
Virginia	8,631,393	255,537 (204,170 - 318,070)	255,551 (204,176 - 318,094)	453,503 (366,041 - 561,173)
Illinois	12,812,508	189,168 (136,556 - 255,786)	310,044 (222,751 - 418,851)	425,530 (311,046 - 568,276)
Kentucky	4,505,836	181,091 (129,273 - 246,659)	181,093 (129,273 - 246,663)	416,316 (308,487 - 551,076)
Massachusetts	7,029,917	224,572 (183,925 - 272,102)	245,226 (198,850 - 300,022)	409,398 (336,032 - 494,949)
Colorado	5,773,714	76,753 (55,081 - 104,864)	102,148 (70,853 - 143,668)	397,688 (300,789 - 519,484)
South Carolina	5,118,425	245,820 (198,666 - 305,570)	245,824 (198,666 - 305,590)	394,621 (320,498 - 487,852)
Georgia	10,711,908	231,076 (183,653 - 290,187)	231,103 (183,670 - 290,223)	393,534 (318,044 - 486,935)
Tennessee	6,910,840	147,857 (110,617 - 195,505)	147,897 (110,626 - 195,580)	386,065 (293,196 - 502,912)
West Virginia	1,793,716	157,742 (102,019 - 228,475)	157,759 (102,031 - 228,502)	383,446 (260,164 - 538,474)
North Carolina	10,439,388	236,306 (180,736 - 308,587)	236,307 (180,736 - 308,588)	383,358 (294,477 - 499,265)
Indiana	6,785,528	164,903 (119,648 - 220,704)	169,897 (122,794 - 227,978)	359,513 (264,282 - 475,767)
New Mexico	2,117,522	118,634 (75,919 - 172,656)	125,478 (78,558 - 186,081)	353,226 (242,475 - 492,265)

Oregon	4,237,256	135,644 (101,496 - 178,618)	158,069 (114,090 - 214,957)	353,124 (266,904 - 462,645)
Mississippi	2,961,279	227,727 (167,956 - 303,852)	227,727 (167,956 - 303,852)	301,315 (223,728 - 399,758)
Alabama	5,024,279	156,795 (114,292 - 211,145)	156,802 (114,298 - 211,158)	292,730 (218,419 - 387,036)
Washington	7,705,281	116,274 (84,646 - 154,854)	178,890 (124,923 - 246,913)	268,417 (193,187 - 362,954)
Connecticut	3,605,944	129,891 (104,710 - 158,171)	145,281 (116,082 - 178,578)	261,915 (211,045 - 319,798)
Idaho	1,839,106	36,866 (27,239 - 48,894)	113,872 (78,424 - 160,419)	254,667 (187,928 - 338,486)
Oklahoma	3,959,353	99,346 (62,089 - 150,385)	108,803 (65,508 - 169,833)	220,869 (148,992 - 319,116)
Kansas	2,937,880	64,813 (37,028 - 105,013)	97,375 (54,200 - 161,442)	215,810 (135,269 - 327,548)
Wisconsin	5,893,718	76,529 (54,440 - 104,923)	80,605 (56,948 - 111,304)	211,509 (156,373 - 281,530)
Missouri	6,154,913	95,376 (64,621 - 137,491)	111,470 (72,588 - 165,341)	194,700 (134,127 - 276,387)
Arkansas	3,011,524	125,085 (89,013 - 172,910)	136,950 (95,908 - 192,002)	190,377 (136,207 - 262,630)
Maryland	6,177,224	79,369 (64,051 - 97,880)	79,369 (64,051 - 97,880)	165,187 (135,737 - 201,028)
Iowa	3,190,369	56,384 (35,178 - 85,084)	56,386 (35,179 - 85,087)	158,904 (102,203 - 233,917)
Minnesota	5,706,494	48,475 (37,880 - 62,768)	89,725 (65,133 - 122,552)	157,837 (118,565 - 209,086)
Nevada	3,104,614	48,897 (33,192 - 68,627)	48,969 (33,218 - 68,776)	147,359 (110,699 - 192,227)
Nebraska	1,961,504	62,986 (39,659 - 95,617)	86,317 (49,357 - 138,035)	144,443 (88,671 - 221,720)
Montana	1,084,225	29,680 (18,448 - 44,694)	48,285 (26,979 - 78,331)	107,709 (66,015 - 165,475)
North Dakota	779,094	32,201 (23,971 - 43,227)	38,085 (27,362 - 52,839)	97,700 (74,099 - 128,071)
Utah	3,271,616	26,566 (20,406 - 34,142)	44,564 (30,846 - 62,280)	95,295 (72,166 - 124,231)
New Hampshire	1,377,529	37,343 (28,364 - 48,793)	40,682 (30,821 - 53,317)	81,794 (62,508 - 106,004)
Wyoming	576,851	10,640 (6,078 - 16,980)	23,786 (12,805 - 39,783)	64,611 (38,925 - 100,043)
South Dakota	886,667	21,859 (13,365 -	26,791 (15,733 -	62,263 (41,447 -

		33,706)	42,757)	91,576)
Maine	1,362,359	19,155 (14,172 - 25,731)	24,947 (17,761 - 34,788)	48,070 (35,096 - 65,449)
Vermont	643,077	15,395 (10,026 - 22,154)	29,194 (18,353 - 43,193)	46,953 (30,027 - 68,828)
Delaware	989,948	33,017 (25,271 - 42,887)	33,017 (25,271 - 42,887)	44,283 (34,061 - 57,264)
Rhode Island	1,097,379	31,707 (22,823 - 42,310)	31,707 (22,823 - 42,310)	43,306 (31,549 - 57,290)
District of Columbia	689,545	4,174 (3,887 - 4,510)	4,174 (3,887 - 4,510)	21,560 (19,942 - 23,390)

Supplementary Table 3. Total housing units in contiguous US states and estimates of total housing units in the SFHA, best-available SFHA, or either SFHA in contiguous US states.

State	Total housing units	Total housing units in SFHA	Total housing units in best-available SFHA	Total housing units in either SFHA
Florida	9,865,350	2,204,642	2,204,657	2,607,006
Texas	11,589,324	600,239	698,599	1,060,804
California	14,392,140	454,477	454,478	994,537
New York	8,488,066	298,223	398,623	565,685
Louisiana	2,073,200	408,987	439,314	545,237
New Jersey	3,761,229	321,498	326,748	407,612
Pennsylvania	5,742,828	147,711	147,711	336,308
Michigan	4,570,173	94,793	108,181	294,773
Ohio	5,242,524	97,010	99,907	260,730
North Carolina	4,708,710	154,175	154,175	239,209
South Carolina	2,344,963	154,984	154,987	231,724
Arizona	3,082,000	67,950	67,950	228,214
Virginia	3,618,247	125,592	125,598	221,932
Massachusetts	2,998,537	126,424	136,991	214,826
Illinois	5,426,429	86,778	143,125	198,645
Colorado	2,491,404	37,193	49,851	192,161
Kentucky	1,994,323	83,845	83,846	191,988
West Virginia	855,635	77,937	77,949	190,108
Georgia	4,410,956	108,259	108,273	182,288
Tennessee	3,031,605	67,306	67,320	176,814
Indiana	2,923,175	78,302	80,683	174,753
Oregon	1,813,747	64,774	75,346	167,316
Alabama	2,288,330	95,715	95,719	165,988
New Mexico	940,859	53,213	57,384	161,269
Mississippi	1,319,945	108,690	108,690	141,474
Connecticut	1,530,197	64,625	72,562	126,543

Washington	3,202,241	53,882	84,227	123,830
Wisconsin	2,727,726	43,175	47,549	121,537
Idaho	751,859	18,281	49,869	112,402
Missouri	2,786,621	51,064	59,448	104,307
Oklahoma	1,746,807	44,428	49,420	103,863
Maryland	2,530,844	49,351	49,351	102,078
Kansas	1,275,689	29,258	43,401	98,935
Arkansas	1,365,265	58,420	64,347	89,220
Minnesota	2,485,558	24,932	52,967	88,435
Iowa	1,412,789	28,450	28,452	78,415
Nebraska	844,278	30,994	42,651	69,788
Nevada	1,281,018	23,654	23,689	65,279
Montana	514,803	15,230	24,802	55,652
New Hampshire	638,795	22,672	26,235	49,381
North Dakota	370,642	15,075	17,754	45,147
Maine	739,072	17,829	24,340	42,869
Delaware	448,735	32,074	32,074	38,555
Utah	1,151,414	10,754	18,765	36,804
Wyoming	271,887	5,424	12,871	33,299
South Dakota	393,375	10,862	12,836	31,054
Vermont	334,318	8,563	16,983	27,065
Rhode Island	483,474	20,147	20,147	26,471
District of Columbia	350,364	3,036	3,036	12,924

Supplementary Table 4. Occupied housing units in contiguous US states and estimates of occupied housing units in the SFHA, best-available SFHA, or either SFHA in contiguous US states.

**State**	**Occupied housing units**	**Occupied housing units in SFHA**	**Occupied housing units in best-available SFHA**	**Occupied housing units in either SFHA**
Florida	8,529,067	1,755,660 (1,470,381 - 1,914,669)	1,755,674 (1,470,392 - 1,914,684)	2,084,138 (1,747,398 - 2,269,959)
California	13,475,623	415,648 (345,623 - 443,077)	415,649 (345,623 - 443,078)	910,133 (764,543 - 966,824)
Texas	10,491,147	508,558 (400,649 - 561,674)	594,742 (464,942 - 657,537)	913,897 (729,717 - 1,000,852)

Louisiana	1,831,610	358,926 (276,344 - 395,903)	383,926 (294,044 - 424,830)	480,100 (371,635 - 528,124)
New York	7,715,172	261,115 (222,812 - 281,970)	335,483 (271,550 - 372,052)	481,392 (388,445 - 532,517)
Pennsylvania	5,210,598	126,480 (88,360 - 144,323)	126,480 (88,360 - 144,323)	291,013 (203,812 - 329,917)
New Jersey	3,426,102	212,339 (161,882 - 263,246)	217,267 (166,090 - 268,434)	283,535 (218,158 - 342,769)
Michigan	4,041,760	76,704 (58,303 - 87,046)	82,998 (61,416 - 96,866)	235,291 (178,744 - 268,295)
Ohio	4,808,773	83,440 (60,793 - 93,460)	86,062 (62,609 - 96,322)	226,814 (168,532 - 251,587)
Arizona	2,705,878	55,021 (40,127 - 63,574)	55,021 (40,127 - 63,574)	192,962 (149,630 - 215,245)
Virginia	3,321,218	107,387 (85,391 - 119,098)	107,392 (85,393 - 119,104)	189,978 (152,264 - 210,547)
Illinois	4,998,395	77,850 (59,410 - 85,168)	126,698 (92,207 - 140,909)	176,541 (131,794 - 194,862)
Kentucky	1,797,937	72,296 (50,832 - 82,387)	72,297 (50,832 - 82,388)	167,373 (120,877 - 188,926)
Massachusetts	2,749,225	100,568 (80,905 - 114,106)	109,653 (87,473 - 124,405)	178,296 (144,993 - 198,443)
Colorado	2,257,815	31,580 (23,870 - 35,540)	41,708 (30,622 - 47,536)	163,806 (129,487 - 180,814)
South Carolina	2,048,912	107,845 (83,505 - 124,807)	107,847 (83,507 - 124,810)	170,711 (133,784 - 194,482)
Georgia	4,020,808	91,571 (70,940 - 102,715)	91,584 (70,946 - 102,729)	156,112 (123,287 - 173,282)
Tennessee	2,742,947	58,346 (43,897	58,354	154,764

		- 65,373)	(43,901 - 65,387)	(118,168 - 171,918)
West Virginia	743,442	64,879 (40,872 - 77,015)	64,889 (40,881 - 77,027)	160,710 (105,171 - 187,897)
North Carolina	4,160,856	101,169 (73,557 - 123,166)	101,169 (73,557 - 123,166)	162,696 (119,020 - 196,668)
Indiana	2,667,542	68,292 (51,584 - 75,513)	70,398 (52,948 - 77,876)	151,821 (114,347 - 167,826)
New Mexico	829,514	46,120 (31,527 - 52,504)	49,190 (33,090 - 56,595)	142,511 (103,149 - 159,171)
Oregon	1,671,983	57,525 (45,638 - 62,607)	66,636 (51,617 - 73,113)	150,477 (120,913 - 162,803)
Mississippi	1,158,193	92,069 (65,372 - 105,023)	92,069 (65,372 - 105,023)	121,011 (86,725 - 137,181)
Alabama	2,011,947	64,879 (45,139 - 76,370)	64,882 (45,140 - 76,374)	122,066 (87,773 - 141,162)
Washington	2,974,692	45,553 (34,831 - 50,498)	70,548 (52,157 - 79,110)	105,325 (80,208 - 116,742)
Connecticut	1,418,069	56,404 (45,547 - 61,813)	63,302 (50,783 - 69,470)	112,238 (91,269 - 122,133)
Idaho	676,206	15,383 (11,777 - 17,163)	42,741 (31,497 - 48,023)	97,397 (74,971 - 107,100)
Oklahoma	1,535,830	37,757 (25,832 - 43,533)	41,546 (27,890 - 48,450)	88,561 (64,377 - 100,459)
Kansas	1,151,360	25,624 (17,332 - 28,966)	37,601 (24,959 - 42,981)	87,705 (61,871 - 97,822)
Wisconsin	2,428,361	35,059 (25,645 - 40,022)	37,091 (26,699 - 43,186)	96,011 (71,119 - 110,422)
Missouri	2,479,146	39,986 (28,506 - 45,831)	46,621 (32,456 -	82,017 (59,126 - 93,765)

			53,925)	
Arkansas	1,199,395	49,679 (35,574 - 56,566)	54,098 (38,049 - 62,246)	75,591 (54,392 - 86,266)
Maryland	2,321,208	34,002 (26,133 - 39,880)	34,002 (26,133 - 39,880)	67,161 (52,624 - 77,804)
Iowa	1,288,560	24,521 (17,403 - 27,495)	24,523 (17,404 - 27,497)	66,557 (46,873 - 74,516)
Minnesota	2,253,990	21,308 (17,269 - 23,433)	40,037 (29,042 - 47,222)	68,783 (51,432 - 79,467)
Nevada	1,177,649	20,841 (14,755 - 23,232)	20,869 (14,767 - 23,267)	58,533 (45,007 - 63,843)
Nebraska	773,312	26,715 (19,530 - 29,862)	36,698 (25,300 - 41,496)	60,902 (43,618 - 67,975)
Montana	447,812	12,828 (8,597 - 14,924)	20,476 (12,877 - 24,355)	45,930 (30,320 - 54,157)
North Dakota	322,553	13,632 (10,727 - 14,779)	15,919 (12,303 - 17,409)	41,060 (32,307 - 44,352)
Utah	1,057,252	9,236 (7,094 - 10,287)	15,439 (10,820 - 17,763)	31,450 (23,522 - 35,225)
New Hampshire	556,357	16,874 (12,362 - 19,921)	18,447 (13,291 - 22,230)	36,102 (26,467 - 42,854)
Wyoming	234,965	4,591 (2,922 - 5,341)	10,284 (6,103 - 12,613)	27,778 (17,880 - 32,613)
South Dakota	350,560	9,089 (6,087 - 10,422)	10,751 (7,063 - 12,395)	26,368 (18,917 - 29,825)
Maine	582,437	9,067 (5,763 - 12,687)	11,826 (7,106 - 17,197)	22,329 (13,948 - 31,126)
Vermont	271,890	6,819 (4,406 - 8,182)	12,578 (7,775 - 15,648)	20,350 (12,880 - 25,192)

Delaware	386,375	14,817 (10,218 - 20,277)	14,817 (10,218 - 20,277)	19,581 (13,858 - 25,783)
Rhode Island	441,274	15,332 (10,629 - 18,743)	15,332 (10,629 - 18,743)	20,877 (14,981 - 24,893)
District of Columbia	312,448	2,121 (1,924 - 2,265)	2,121 (1,924 - 2,265)	11,149 (10,215 - 11,735)

Parcel classifications for validation

Local parcel boundaries with land use classifications for Mecklenburg County, NC, Miami-Dade County, FL, and Sacramento County, CA were used to validate the generated building footprint classifications. Single value land uses (i.e., not mixed land use) were sorted into "residential" or "not residential" (Supplementary Table 5). Mixed or ambiguous land uses were classified as "unclassified" and were not included in the validation workflow. The Mecklenburg County data contained multiple polygons for each parcel, so the dataset was "flattened" and the "most residential" land use was preserved. Miami-Dade County parcel data was joined to spatially-generalized land use data to create parcels with land uses. Sacramento County, CA parcel data already contained the necessary land use information and was not modified.

Supplementary Table 5. Classification of local parcel datasets.

**Location**	**Residential land uses**	**Non-Residential land uses**
Mecklenburg County, NC	Condo/Townhome, Multi-Family, Single-Family	Commercial, Govt-Inst, Hotel/Motel, Office, Warehouse
Miami-Dade County, FL	Residential, Single-Family (Med. Density), Single-Family (High Density), Townhouses, Single-Family (Low Density), Two-Family (Duplexes), Low-Density Multi-Family, Multi-Family (Migrant Camps), Mobile Home Parks, Nursing Homes	Agriculture, Airports/Ports, Cemeteries, Coastal Waters, Commercial, Communications/Utilities/Terminals/Plants, Expressway, Industrial, Industrial Extraction, Inland Water, Institutional, Office, Parks and Recreation, Shopping Centers/Commercial/Stadiums/Tracks, Streets/Roads/Expressways/Ramps, Streets/Roads/Canals, Transient-Residential (Hotels/Motels), Transportation/Communication/Utilities, Water
Sacramento County, CA	Residential	Agricultural, Care/Health, Church/Welfare, Industrial, Miscellaneous, Office, Public/Utilities, Recreational,

		Retail/Commercial

Duplex building footprints and housing units

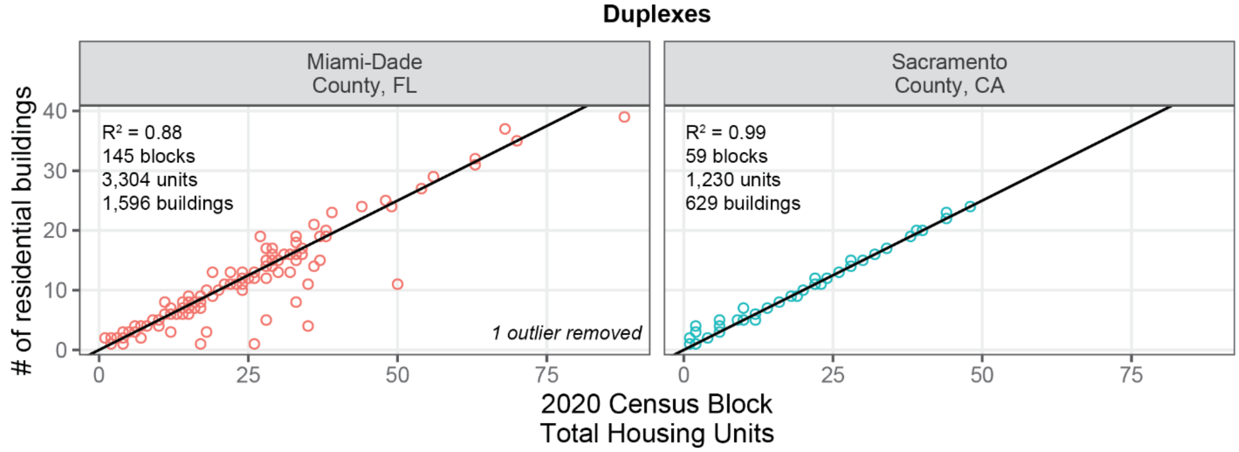

Supplementary Figure 1. Comparison of the estimated number of residential buildings from building footprints and total housing units for census blocks containing only “duplex” (two-family) residential parcels.

Share of residential buildings that are single-family

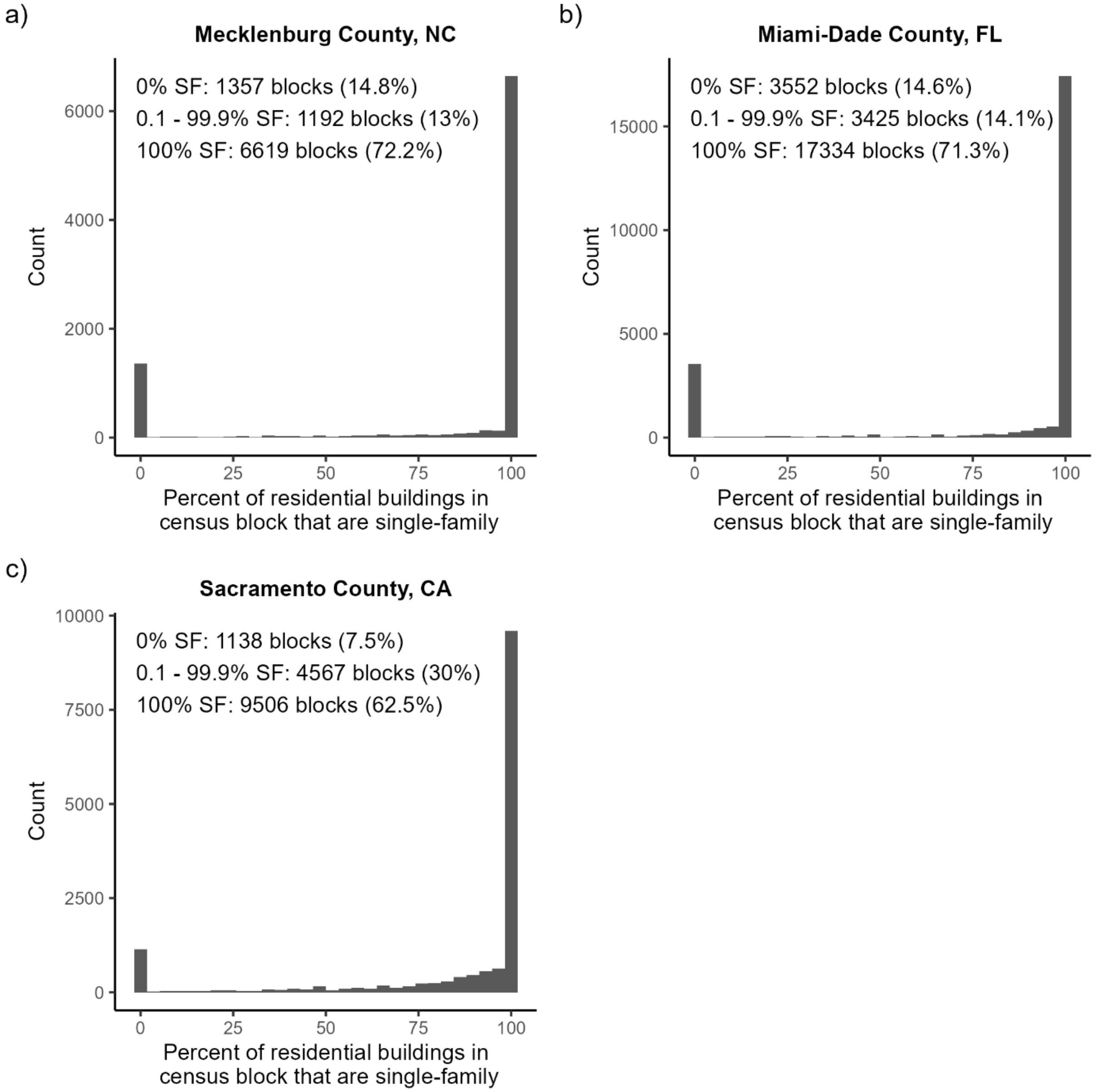

Supplementary Figure 2. Histograms showing the count of census blocks based on the percent of residential buildings that are single-family in each validation location.
